# Automated chest screening based on a hybrid model of transfer learning and convolutional sparse denoising autoencoder

**DOI:** 10.1186/s12938-018-0496-2

**Published:** 2018-05-23

**Authors:** Changmiao Wang, Ahmed Elazab, Fucang Jia, Jianhuang Wu, Qingmao Hu

**Affiliations:** 1 Shenzhen Institutes of Advanced Technology, Chinese Academy of Sciences, 1068 Xueyuan Boulevard, Shenzhen, 518055 China; 2 University of Chinese Academy of Sciences, 52 Sanlihe Road, Beijing, 100864 China; 3 Guangdong Key Laboratory for Biomedical Measurements and Ultrasound Imaging, School of Biomedical Engineering, Health Science Center, Shenzhen University, Shenzhen, 518060 China; 4 Department of Computer Science, Misr Higher Institute for Commerce and Computers, Mansoura, 35516 Egypt; 5 Key Laboratory of Human-Machine Intelligence Synergy Systems, 1068 Xueyuan Boulevard, Shenzhen, 518055 China

**Keywords:** Chest screening, Computer aided diagnosis, Deep learning, Autoencoder, Receiver operating characteristic

## Abstract

**Objective:**

In this paper, we aim to investigate the effect of computer-aided triage system, which is implemented for the health checkup of lung lesions involving tens of thousands of chest X-rays (CXRs) that are required for diagnosis. Therefore, high accuracy of diagnosis by an automated system can reduce the radiologist’s workload on scrutinizing the medical images.

**Method:**

We present a deep learning model in order to efficiently detect abnormal levels or identify normal levels during mass chest screening so as to obtain the probability confidence of the CXRs. Moreover, a convolutional sparse denoising autoencoder is designed to compute the reconstruction error. We employ four publicly available radiology datasets pertaining to CXRs, analyze their reports, and utilize their images for mining the correct disease level of the CXRs that are to be submitted to a computer aided triaging system. Based on our approach, we vote for the final decision from multi-classifiers to determine which three levels of the images (i.e. normal, abnormal, and uncertain cases) that the CXRs fall into.

**Results:**

We only deal with the grade diagnosis for physical examination and propose multiple new metric indices. Combining predictors for classification by using the area under a receiver operating characteristic curve, we observe that the final decision is related to the threshold from reconstruction error and the probability value. Our method achieves promising results in terms of precision of 98.7 and 94.3% based on the normal and abnormal cases, respectively.

**Conclusion:**

The results achieved by the proposed framework show superiority in classifying the disease level with high accuracy. This can potentially save the radiologists time and effort, so as to allow them to focus on higher-level risk CXRs.

## Background

Chest screening is a basic procedure in radiology for lung disease prediction and diagnosis. However, such diagnosis is very time consuming and subjective. The turnaround time is of great importance in radiology as it is an important criterion to evaluate radiologists rather than the quality of their reports [1]. Especially in rural areas, direct care providers rely mainly on teleradiology for their chest X-rays (CXRs) interpretation. The emphasis on turnaround time can result in sub-standard reports, confusion, misdiagnosis, and gaps in communication with primary care physicians. All of these can severely and negatively impact patients’ care and may have life-changing consequences for patients. Our work is inspired by the recent progresses in image classification and segmentation. The former has substantially improved performance, largely due to the introduction of ImageNet database [2] and the advances in deep convolutional neural networks (CNNs) that effectively helps to recognize the images with a large pool of hierarchical representations. The CNNs can be used successfully in medical image classification and segmentation [3–6]. Many other techniques on lung disease detection and classification have been proposed [7–19].

Sufficient performance, no increase in reading time, seamless workflow integration, regulatory approval, and cost efficiency are the key points to the radiologists [20]. Tataru et al. applied three different neural network models for abnormality detection in CXR images that are not public released [21]. Our work will focus on classifying the CXRs as normal or abnormal in order to assist radiologists to move more quickly and efficiently. We then use the features extracted from deep neural network model for the classification of abnormalities in the CXRs. That is to say, we will classify the CXRs at three status levels: obvious abnormal, obvious normal, and uncertainty. Furthermore, a large collection of medical images can be automatically classified to three main levels, and the uncertainty status level images can be checked carefully by radiologist. A computer-aided triage system can mitigate these issues in several ways. Firstly, it will allow radiologists to focus their attention immediately on higher-risk cases. Secondly, it will provide radiologists with more information to help them correct potential misdiagnoses. The CXRs input to our algorithm will be a digital image format along with a label stating ‘normal’ or ‘abnormal’.

In recent years, many methods were proposed for CXRs classification. There are many conventional machine learning methods to study the classification of X-ray chest radiographs [22, 23], such as based on texture and deformation features [24] or ensemble methods [25]. In addition, with the development of deep learning technology, many deep learning method are also applied to the field of medical image analysis. Yao et al. [26] explored the correlation among the 14 pathologic labels based on global images in Chest X-ray 14 [27] in order to classify CXRs, and rendering it as a multi label recognition problem. Using a variant of DenseNet [28] as an image encoder, they adopted the long-short term memory networks (LSTM) [29] so as to capture dependencies. Kumar et al. [30] investigated that which loss function is more suitable for training CNNs from scratch and presented a boosted cascaded CNN for global image classification. CheXNet [31] is the recent effective method for fine-tuning a 121-layer DenseNet on the global chest X-ray images, which has a modified last fully-connected layer.

In this work, we demonstrate how to automatically classify CXRs at different status levels. Four publicly available radiology datasets that contain CXR images and reports namely are exploited as follows: the OpenI [17] open source literature, JSRT [32], Shenzhen Chest X-ray set [33], and NLM-Montgomery County Chest X-ray set [33]. A common challenge in medical image analysis is the data bias. When considering the whole population, diseased cases are much less than healthy cases, which is also the case in the CXRs datasets used. In our study, normal cases account for 75.9% (1883 images) of the entire dataset (2480 images), and the abnormal cases account for 24.1% (597 images) of the entire dataset.

Inspired by the ideas introduced in [34, 35], we employ the already trained models to obtain the high level features and use them to infer the probability of image level. Then, we train an autoencoder network with the obtained joint image and generate new image based on decoder network to obtain the reconstruction error. With the combined metrics including the probability and reconstruction error based on ROC curve, we can achieve a score for classifying the image level. Finally, we vote for the decision of the image level using the ensemble method. We can ultimately generate the better and more accurate image level. It will greatly reduce the workload of medical experts and assist them to pay more attention to the suspected higher risk chest X-rays.

The outline of this paper is organized as follows. In “Methods”, we present the preprocessing of the steps for the acquired CXR images, and features extraction is presented by transfer learning using a classical deep model. Moreover, the convolutional sparse denoising autoencoder network to reconstruct the image is proposed and image classification with CNNs, which are fine-tuned with extracted features. We report the experimental results and discussions in “Results” and “Discussion”, respectively. Finally, conclusions and the future work are summarized in “Conclusion”.

## Methods

This section discusses the datasets, preprocessing, lung segmentation, feature extraction, and classification steps. The convolutional sparse denoising autoencoder (CSDAE) is proposed to get the reconstruction error for classifying CXRs. Moreover, image classification with CNNs can allow us to obtain the probability in order to classify CXRs. Finally, the CXRs are classified by combining predictors based on the ROC curves.

### Datasets

To ensure the robustness of our method, we test the proposed technique on a total of 2480 images including 1883 negative cases and 597 positive cases from four public datasets, namely JSRT, OpenI, SZCX, and MC. The data is randomly split into 70% for training set and 30% validation set. The number of positive cases and negative cases in the training set and validation set are summarized in Table 1.

**Table 1:** Number of positive cases and negative cases used in training and validation sets

Training set		Validation set	
Positive	Negative	Positive	Negative
418	1318	179	565

Next, we give detailed description of every datasets as follows.

#### Japanese Society of Radiological Technology (JSRT)

A standard database that is acquired from the JSRT. The detailed information of JSRT can be assessed in [32]. This dataset contains 154 cases with confirmed lung nodules by CT and 93 normal cases without nodules. The images have been digitized to 12-bit based on 2048 × 2048 pixels and at a resolution of 0.175 mm per pixel. The nodule size ranges from 5 to 40 mm in diameter.

#### OpenI

Generally, we examine the posteroanterior views through the chest of the subject from back to front. Hence, we only select the frontal images (numbering 3812) in OpenI dataset that has totally 7470 images including 3658 as lateral images. Only 1433 images including 49 cases with nodule and tuberculosis, and 1384 normal cases are selected from the frontal images. The images have following different resolutions (in pixels): 512 × 624, 512 × 420, and 512 × 512.

#### China set—The Shenzhen set—chest X-ray database (SZCX)

The standard digital image database for Tuberculosis is created by the National Library of Medicine, Maryland, USA, in collaboration with Shenzhen No. 3 People’s Hospital, Guangdong Medical College, Shenzhen, China [33]. The Chest X-rays are from out-patient clinics and had been captured as part of the daily routine using Philips DR Digital Diagnose systems. There are 336 cases with manifestation of tuberculosis and 326 normal cases. The format of this dataset is PNG where the image size varies for each X-ray with approximately 3000 × 3000 pixels. This set includes some pediatric images. As a result, the image sizes in the dataset vary: image width: minimum is 1130 pixels and maximum is 3001 pixels; for the image height, the minimum is 948 pixels and the maximum is 3001 pixels. The resolutions range from 1024 × 1024 to around 2480 × 2480 pixels. The dataset is publicly available in: http://archive.nlm.nih.gov/repos/chestImages.php.

#### Montgomery-County chest X-ray database (MC)

The standard digital image database for Tuberculosis is created by the National Library of Medicine in collaboration with the Department of Health and Human Services, Montgomery County, Maryland, USA [33]. This image set contains data from X-rays collected under Montgomery County’s Tuberculosis screening program. There are 58 cases with manifestation of tuberculosis and 80 normal cases. Its format is PNG and the matrix size is 4020 × 4892 or 4892 × 4020 pixels. The pixel spacing in vertical and horizontal directions is 0.0875 mm while the number of gray levels is 12 bits. The dataset is publicly available in: http://archive.nlm.nih.gov/repos/chestImages.php.

### Preprocessing

Preprocessing is a preliminary stage in designing computer aided diagnosis (CAD) systems. Its main goal is to enhance the characteristics of the image that can help to improve performance in subsequent stages. There are many sources of variance in the CXRs data, which may negatively affect the performance of downstream classification tasks using feature-based methods or neural networks. We first process all images based on histogram equalization in order to increase contrast within each CXRs image. After that, we label images that have disease as positive cases, and all other normal image as negative cases. We randomly split the entire dataset into 70% for training and the remaining 30% for testing. Before inputting the image into the network for extraction of the high-level features, we crop the region from the original image, and then down-sample every image to a 512 × 512 matrix of pixels.

**Fig. 1:**
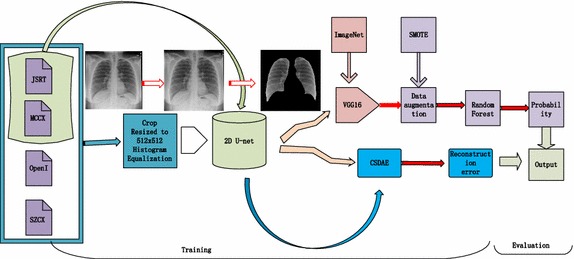
Workflow diagram of our proposed method

Our chest screening system consists of several modules in the processing pipeline (Fig. 1). The first module aims to perform preprocessing on the chest radiographs. Due to the high effect of the outside of lung, we segment the region of lung using U-net [36] and achieve the promising dice and intersection-over-union (IoU) in the second module. High level features are extracted using transfer learning from pre-trained deep network model in the third module, which are used for training the classifier so as to give the confidence of the status level pertaining to the image. The fourth module is to reconstruct the image with the CSDAE. Finally, the two metric indices are used to determine the final decision on the level of the image.

The development of such systems has so far been hindered by immense variance of CXRs images and lack of labeled data. The normal versus abnormal ratio is 3.15:1. To reduce the impact of the data imbalance, we augment the data by using SMOTE [37], SMOTE-TK [38], and SMOT-ENN [38] in the feature space. Finally, we normalize the images based on their mean and standard deviation in the training feature set. In addition, we implement the data augmentation by a rotation range from 0 to 10, with a width shift and a height shift of both 0.2 for each training image.

### Lung field segmentation

**Fig. 2:**
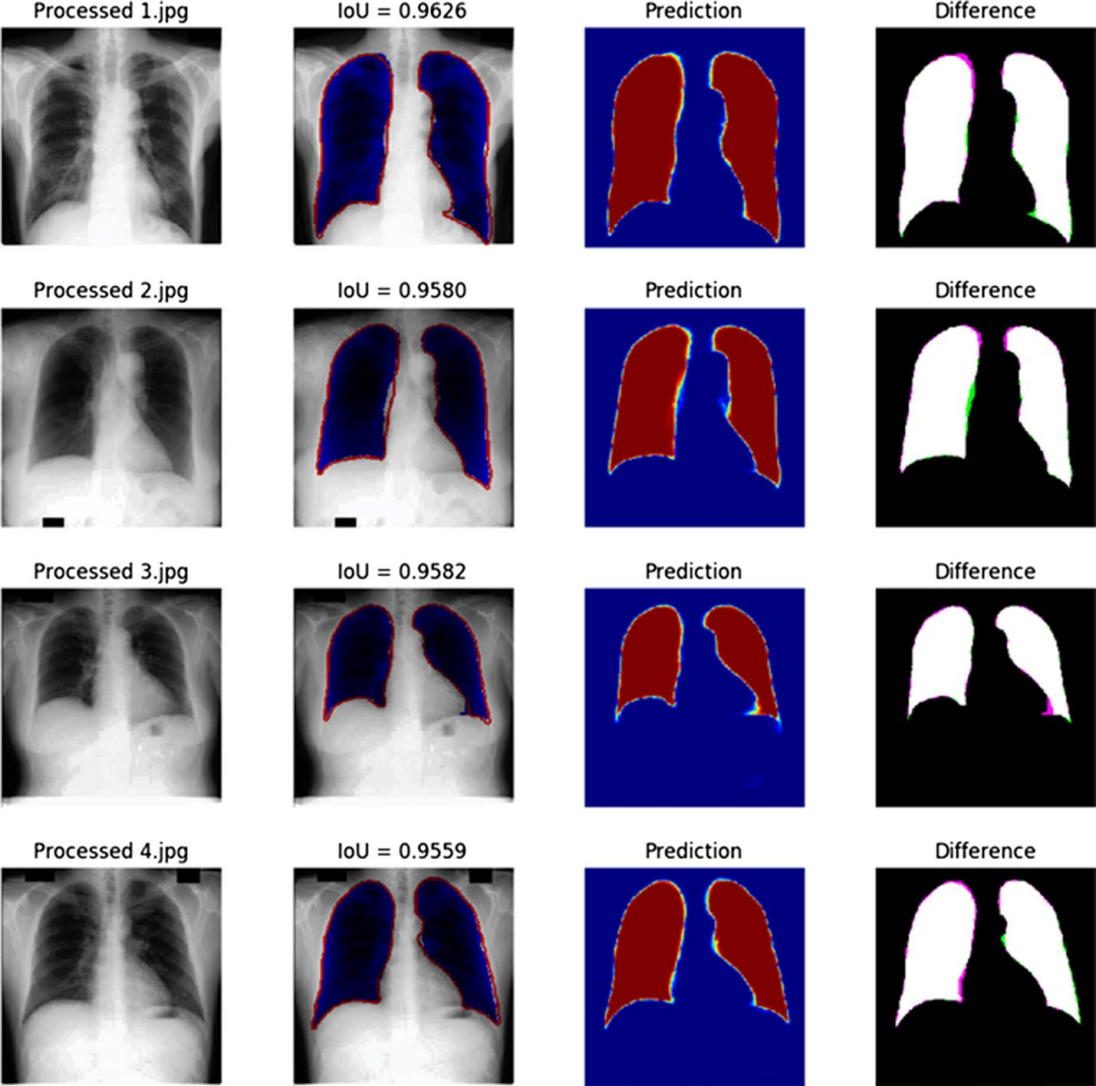
Segmentation of lung from public database based on U-net

The key step in CAD scheme for lung disease detection in chest radiographs is the segmentation of the lung field whereby many approaches have been proposed. All images in the utilized datasets contain regions outside lungs that are not relevant to lesion detection. To diminish the risk that the features present in the images, but are irrelevant to lesion detection tends to distort the final results, we decide to segment the lung regions of the CXRs. The most classic method is a graph cut based segmentation method that is mentioned in [39]. It begins with content based image retrieval using a training set along with its defined masks. The initial specific anatomical model is created using SIFT-flow for deformable registration of training masks for the patient CXR. Finally, a graph cuts optimization procedure with a custom energy function is used. These methods can be broadly classified into four categories: (1) rule-based techniques, (2) pixel classification technique, (3) deformable model-based techniques, and 4) hybrid techniques [39]. Although these methods yield accurate segmentation based on pixel classification, they can result in non-desirable shapes, such as in [40, 41]. Recently, there has been an increasing interest towards the exploration of deep learning methods. There are many works on image segmentation employing these method such as references [36, 42–44]. The encoder-decoder structure of U-net can capture the delicate boundaries of objects by exploiting the high resolution feature maps in the decoder path. Hence, we can employ the U-net to account for the high variability of lung shapes with the JSRT and MC. Figure 2 shows examples of the final boundaries of the lung areas with high IoU 0.963 and Dice 0.978. After the segmentation of the lung region, the resulting image is cropped to the size of a minimum bounding box containing all the pixels of the lungs.

### Feature extraction

Feature extraction is another critical component in the CAD systems, which has a great influence on their performance. Recently, deep neural network has gained popularity because of its ability to learn mid and high-level image features. The deep learning method has been applied in many medical image analysis tasks [10, 45, 46] and remarkable results have been achieved. Deep learning methods are more powerful when applied to large training sets. Meanwhile, we can ideally train a CNNs by a large number of medical datasets and achieve promising performance. However, the large datasets in the medical field are usually rare and very expensive to obtain. That is to say, we do not have enough medical data, and deep learning methods are most effective when applied to networks with large number of training data in order to train the deep neural network. However, we can extract the high level features by using deep learning method based on non-medical learning [47]. Recently, many papers have been published in the general computer vision literature using transfer learning, which is an efficient way to utilize image representations learned with CNNs on large-scale annotated datasets. In particular, these are the target domains in which limited data exists. Transfer learning from pre-trained ImageNet can be useful in medical images [7, 35].

In the computer vision domain, large image sets exist (e.g. ImageNet) which enable better training of popular CNNs. In many image recognition tasks pertaining to the large scale visual recognition challenge of ImageNet, a few examples of such CNNs are: Decaf (2014), AlexNet (2012), VGG (2014), Overfeat (2013), Google Inception Net (2015), and ResNet (2016) [48–54]. The CNNs were able to extract improved representations from raw data without requirement for domain knowledge. This was done with no hyper-parameter tuning, which suggests that there are further improvements that can be made. This is important for the task generally as it can mean that there is potential in using CNNs or other deep learning strategies as a “*black box*”, whereby we will be able to achieve excellent machine learning performance neglecting the need of expert-designed feature extraction or domain knowledge.

The VGGNet in [50] was trained over a subset of images from ImageNet containing 1000 categories and 1.2 million images. This network is characterized by its simplicity, as it involves using only 3 × 3 convolutional layers stacked on top of each other with increasing depth. Reducing volume size is handled by max pooling. Two fully-connected layers, each with 4096 nodes is then followed by a softmax classifier. For the fully-connected layers, each has 4096 neurons. We extracted the feature from the fully-connected layer on the pre-trained VGGNet model by the transfer learning to train our classifier. The dataset can be benefited from more complex GoogLeNet [51], which is arguably the current state-of-the-art CNN architectures. However, there are still other classical deep neural networks such as InceptionNet [48] and residual networks [49].

In this paper, we extract the feature using transfer learning from VGGNet16. After that, training the traditional classifier is done using a 10-fold cross validation. Besides, we also design the neural network to fine-tune it from the extracted features.

### CSDAE

An autoencoder (AE) neural network is an unsupervised learning algorithm that applies backpropagation, and setting the target values to be equal to the inputs. In other words, it is trying to learn an approximation to the identity function so that the output can be similar to the input. The AE mostly aims at reducing feature space in order to distill the essential aspects of the data versus more conventional deep learning, which expands the feature space significantly in order to capture non-linearity and subtle interactions within the data. Autoencoder can also be seen as a non-linear alternative to principal component analysis. This trivial function seems not to be very exciting at all; however, if we consider some constraints on the AE, one can discover suitable features for a learning problem in an automatic way. The goal of the AE is to learn a latent or compressed representation of the input data by minimizing the reconstruction error between the input at the encoding layer and its reconstruction at the decoding layer.

**Fig. 3:**
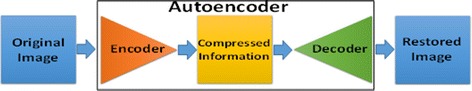
Flow chart of Autoencoder

The AE comprises two parts: the encoder and decoder. The encoder reduces the dimensions of input data so that the original image is compressed. The decoder restores the original image from the compressed data. The autoencoder is a neural network that learns to encode and decode automatically, which can be shown in Fig. 3.

**Fig. 4:**
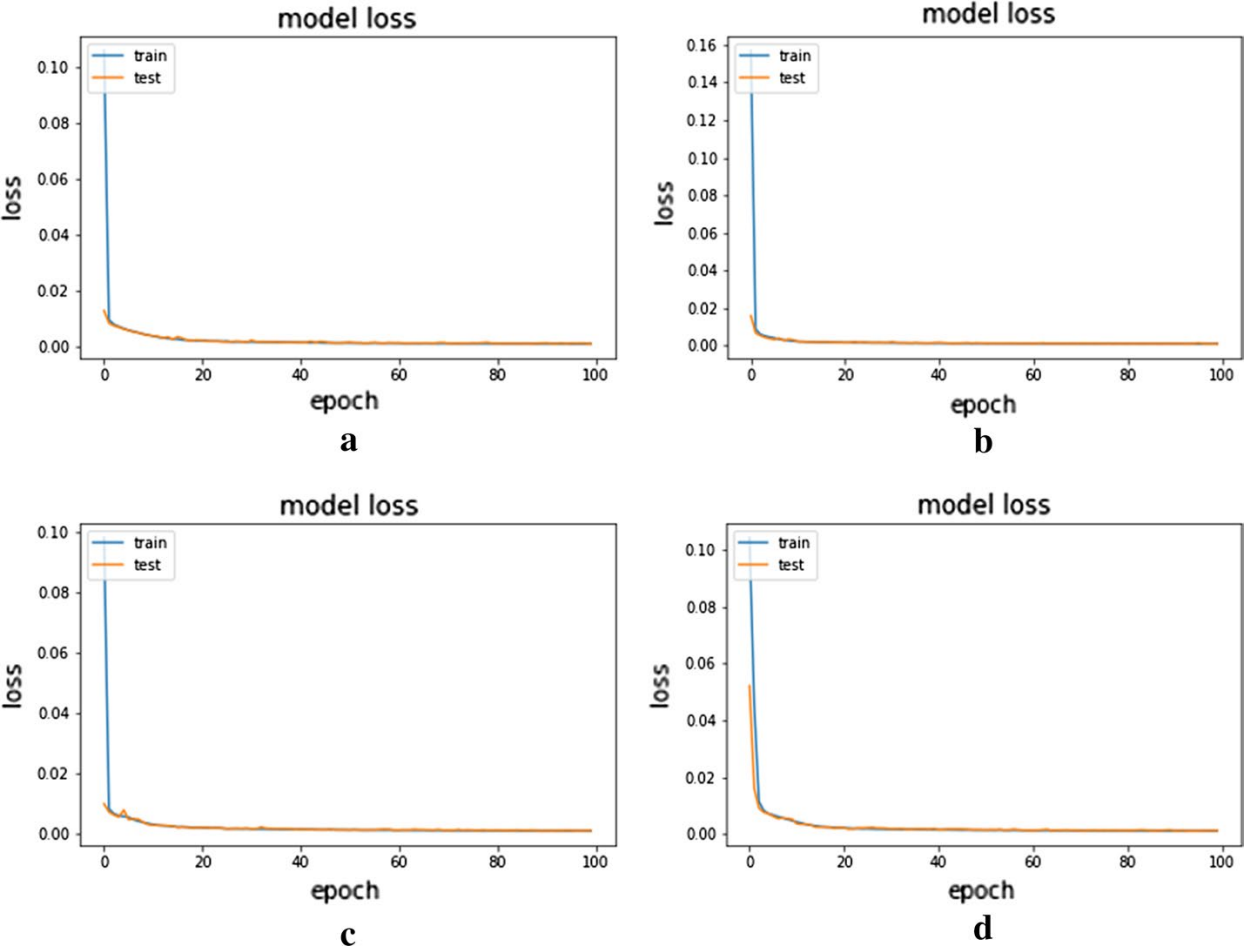
Loss over the epochs on the AE and CSDAE. **a** AE with noise factor 0.01, **b** CSDAE with noise factor 0.01, **c** AE with noise factor 0.05, and **d** CSDAE with noise factor 0.05

Beyond simply learning features by AE, there is a need for reinforcing the sparsity of weights and increasing its robustness to noise. Ng et al. introduced the sparse autoencoder (SAE) [55], which is a variant of the AE. Sparsity is a useful constraint when the number of hidden units is large. SAE has very few neurons that are active. Sparse feature learning is a common method for compressed feature extraction in shallow encoder-decoder-based networks, i.e. in sparse coding [56–59], in AE [60], and in Restricted Boltzmann Machines (RBM) [61, 62]. There is another variant of AE called the denoising autoencoder (DAE) [63], which minimizes the error in reconstructing the input from a stochastically corrupted transformation of the input. The stochastic corruption process involves randomly setting some inputs to zero. The purpose of this denoising autoencoder is to take a noisy image as input and return a clean image as output. In our research, we consider CSDAE to train a convolutional sparse AE, which can reconstruct the input data from a corrupted version by manual addition with random noise (Table 2). Based on our case, Gaussian noise is added to the original image. This approach can effectively integrate the advantages in SAE, DAE, and CNN. This hybrid structure forces our model to learn more abstract and noise-resistant features, which will help to improve the model’s representation learning performance. We reconstruct the original dataset using the reduced set of features and compute the means squared error for both of them (Fig. 4).

**Table 2:** Network architecture of CSDAE

Layer (type)	Output shape	Param#
Input_1(InputLayer)	(None, 512, 512, 1)	0
Conv2d_1(Conv2D)	(None, 512, 512, 16)	160
Max_pooling2d_1(MaxPooling2D)	(None, 256, 256, 16)	0
Conv2d_2(Conv2D)	(None, 256, 256, 8)	1160
Max_pooling2d_2(MaxPooling2D)	(None, 128, 128, 8)	0
Conv2d_3(Conv2D)	(None, 128, 128, 8)	584
Max_pooling2d_3(MaxPooling2D)	(None, 64, 64, 8)	0
Conv2d_4(Conv2D)	(None, 64, 64, 8)	584
Up_sampling2d_1(UpSampling2D)	(None, 128, 128, 8)	0
Conv2d_5(Conv2D)	(None, 128, 128, 8)	584
Up_sampling2d_2(UpSampling2D)	(None, 256, 256, 8)	0
Conv2d_6(Conv2D)	(None, 256, 256, 8)	1168
Up_sampling2d_3(UpSampling2D)	(None, 512, 512, 16)	0
Conv2d_7(Conv2D)	(None, 512, 512, 1)	145

The identity function seems particularly trivial to be learned. However, by placing constraints on the network via limiting the number of hidden units, we can discover interesting structures pertaining to the data. In this paper, we employ convolutional sparse autoencoder in order to reconstruct the original image. The power of CSDAE lies in the form of reconstruction-oriented training, where the hidden units can conserve the efficient feature to represent the input data. Feature extractors are learned by minimizing the reconstruction error of the cost function in [1]. The first term in the cost function is the error term. The second term is a regularization term that is given by

1 $$L\left( {X,Y} \right) = \frac{1}{2}\mathop \sum \limits_{i = 1}^{M} x^{i} - y^{i} + \frac{\lambda }{2}W^{2} ,$$

where X and Y represent the training and reconstructed data while $\lambda$ and $W$ are the regularization parameter and weight, respectively.

In order to obtain a better representation, we consider a rectified linear unit (ReLU) activation function, and the default hyperparameters settings are as follows: learning rate = 0.0001, and batch size = 62. These hyperparameters are chosen as those previously optimized on ImageNet. We set the L1 regularization parameter as equal to 0.00001 in order to determine the sparseness value. The Gaussian noise factors are set to 0.01 and 0.05.

The key idea of CSDAE is to learn a sparse but robust bank of local features. After that, we can compute the reconstruction error by performing an input image subtraction of the reconstructed image from the CSDAE network.

Totally, a 2480 images from the four public databases are used for the experiments, which are randomly split into 70% training (1736 images) and 30% testing (744 images). Only 1318 images were used for training the CSDAE.

### Image classification with CNN

Even when we balance the dataset by augmenting many diseased samples, it is still difficult for CNN to learn a good model to distinguish many abnormal cases from normal cases, which have many variations on their original samples. Therefore, we attain the features from VGGNet16 with pre-training on ImageNet to fine-tune our CNNs with batch-normalization, data-dropout, which is assessed by the cross entropy and focal loss [64]. Default hyperparameter settings are as follows: learning rate = 3e−5, regularization is L1, batch size = 62, and drop out = 0.25 or 0.5. These parameters are chosen as optimized on training data. Table 3 illustrates the network architecture. For the focal loss function, we set lambda = 0.1, and gamma = 0.1. In addition, we train our network on the original training data and other three data augmentation.

**Table 3:** Network architecture of proposed image classification

Layer (type)	Output shape	Param#
Input_4(InputLayer)	(None, 512)	0
Batch_normalization_4(Batch)	(None, 512)	2048
Dropout_4(Dropout)	(None, 512)	0
Dense_4(Dense)	(None, 512)	262,656
Batch_normalization_5(Batch)	(None, 512)	2048
Dropout_5(Dropout)	(None, 512)	0
Dense_5(Dense)	(None, 512)	262,656
Batch_normalization_6(Batch)	(None, 512)	2048
Dropout_6(Dropout)	(None, 512)	0
Dense_6(Dense)	(None, 1)	513

### Combining predictors for classification based on ROC curve

We can attain two metrics from the prediction of CNN and the reconstruction error from the CSDAE. How to combine multiple predictors into a score is the key to the final decision. We assume that the predictors $(C_{1} , C_{2} , \ldots C_{P} )$ are given and the statistical problem is to estimate $\beta = (\beta_{2} , \ldots , \beta_{P} )$ from data in Eq. (2). We seek estimators that are consistent under the risk score model [3], since under that model $f_{\beta } (C)$ is the optimal combination as follows:

2 $$f_{\beta } \left( C \right) = C_{1} + \beta_{2} C_{2} + \cdots \beta_{P} C_{P} .$$

Under a particular circumstance we can allow $f_{\beta } (C)$ to be the “right” combination score for classification based on C. If the risk score is some monotone increasing function of $f_{\beta } (C)$, then we have

3 $$P\left[ {y = 1 |C} \right] = g\left( {C_{1} + \beta_{2} C_{2} + \cdots \beta_{P} C_{P} } \right) = g\left( {f_{\beta } (C)} \right).$$

It follows the Neyman-Pearson lemma [65, 66] that rules based on $f_{\beta } \left( C \right) > c$ are optimal. Assuming only the generalized linear model [3], the optimality of $f_{\beta } (C)$ implies that the ROC curve for any other function of C cannot be higher at any point than the ROC curve for $f_{\beta } (C)$. The area under the ROC curve (AUC) is the most popular ROC summary index. The optimal ROC curve has maximum AUC so we can use it as the basis for an objective function of the data to estimate $\beta$. It is easy to show that the AUC of the empirical ROC curve is the Mann–Whitney U statistic as follows:

4 $$\widehat{AUC}\left( b \right) = \frac{{\mathop \sum \nolimits_{i = 1}^{{n_{D} }} \mathop \sum \nolimits_{i = 1}^{{n_{{\bar{D}}} }} I\left[ {L_{b} (C_{Di} ) > L_{b} (C_{{\bar{D}i}} )} \right]}}{{n_{D} n_{{\bar{D}}} }}.$$

where $n_{D}$ is the number of positive cases and $n_{{\bar{D}}}$ is the number of negative cases. We present the corresponding AUC based estimator of $\beta$ as

5 $$\widehat{\beta }^{AUC} = argmax \left( {\widehat{AUC}(b)} \right).$$

## Results

All experiments are conducted on HP Z840 platform with the Tesla K40c and Quadro K5200, CPU E5-2650 v3 2.30 GHz, memory 126G, and Ubuntu 16.04 operating system.

In total, 2480 images from the four public databases are used in our experiments, which are randomly split into 70% training (1736 images) including 1318 negative cases and 418 positive cases, and 30% testing (744 images) including 565 negative cases and 179 positive cases. The testing data is strictly independent to the training data, which is not used to tune our algorithm. We employ four commonly used metrics to quantitatively evaluate the performance of our method namely, precision, recall, F1, and AUC scores.

**Fig. 5:**
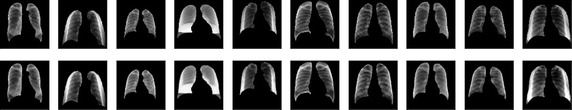
Original lung image and the reconstructed lung image from AE: upper row is the original image while the bottom row is the reconstruction image

**Fig. 6:**
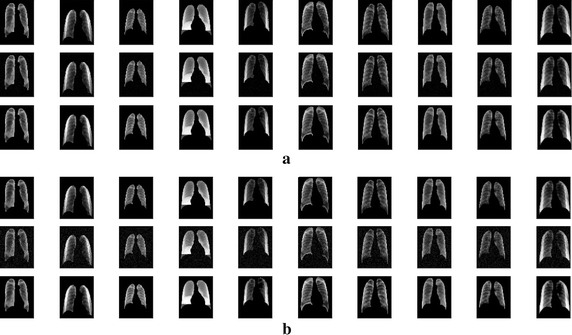
Original lung image and reconstructed lung image from CSDAE. **a** Results of CSDAE with noise factor 0.01: the upper row is the original image, middle row is the image with noise, and bottom row is the reconstructed image. **b** Results of CSDAE with noise factor 0.05: the upper row is the original image, middle row is the image with noise, and bottom row is the reconstructed image

**Fig. 7:**
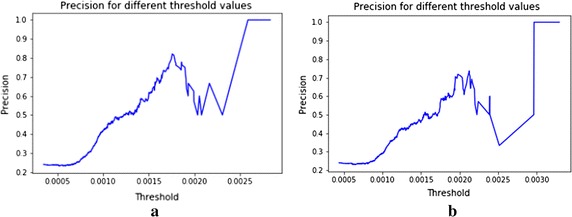
Precision for different threshold in CSDAE (**a**) with noise factor 0.01 and (**b**) noise factor 0.05

Through experiments, we are able to show that these factors elevate the classification accuracy of our CSDAE. They are all indispensable to our model as there are usually a small drop in accuracy when removing these structures. We design a CSDAE network by adding different noise factors of 0.01 and 0.05 for all data. Then, the CSDAE network and the regular AE network are trained and tested on the datasets. The mean and variance metrics of the MSE are used for evaluation, respectively. The experimental results show that our CSDAE network is better than the conventional AE network under different noise levels, which illustrate the reliability of our network design. The detailed comparisons are shown in Table 4. The training process of AE and CSDAE based on different noise factors is presented in Fig. 5. The original lung image and the reconstructed lung image from AE are shown in Fig. 6. Meanwhile, Fig. 7 shows the original lung image and the reconstructed lung image from CSDAE.

**Table 4:** Comparisons of the mean and std of MSE between AE and CSDAE

	Noise factor	Train data MSE (mean ± std)	Test data MSE (mean ± std)	Test noised data MSE (mean ± std)
AE	0.01	0.000983 ± 0.000304	0.001088 ± 0.0004	0.001112 ± 0.000405
CSDAE	0.01	0.000829 ± 0.000282	0.000923 ± 0.000369	0.000922 ± 0.000368
AE	0.05	0.001039 ± 0.000334	0.001146 ± 0.000427	0.001255 ± 0.000445
CSDAE	0.05	0.001003 ± 0.000334	0.001103 ± 0.000421	0.001174 ± 0.00045

Tables 5 and 6 provide the comparison of different classifiers based on four datasets: (1) without data augmentation, (2) smote augmentation (SMOTE), (3) only data augmentation for positive data in training data (positive augmentation), and (4) data augmentation 4 times for training data (4× augmentation). Table 6 shows that the respective performance by CNN with different loss function and data augmentation methods. It demonstrates that the focal loss is useful for the imbalanced dataset compared to when using the cross-entropy loss in Table 6. From the result of Table 5, it shows that CNN classifier has superior performance when compared to the traditional classifier.

**Table 5:** Performance results based on test data using four classifiers: KNN, logistic regression, SVM, random forest

Classifier	Data augmentation	Precision	Recall	F1	AUC
KNN	Without augmentation	0.73	0.58	0.64	0.753
		0.87	0.93	0.9	
	SMOTE	0.44	0.84	0.58	0.752
		0.93	0.67	0.78	
	Positive augmentation	0.57	0.68	0.62	0.758
		0.89	0.68	0.87	
	4× augmentation	0.57	0.84	0.62	0.758
		0.89	0.89	0.87	
Logistic	Without augmentation	0.76	0.54	0.63	0.744
		0.87	0.95	0.91	
	SMOTE	0.61	0.82	0.64	0.79
		0.92	0.76	0.84	
	Positive augmentation	0.57	0.66	0.61	0.754
		0.89	0.84	0.86	
	4× augmentation	0.63	0.72	0.67	0.792
		0.91	0.87	0.89	
SVM	Without augmentation	0.63	0.73	0.68	0.798
		0.91	0.86	0.89	
	SMOTE	0.63	0.77	0.69	0.813
		0.92	0.86	0.89	
	Positive augmentation	0.59	0.64	0.61	0.75
		0.88	0.86	0.87	
	4× augmentation	0.61	0.71	0.66	0.784
		0.9	0.86	0.88	
Random forest	Without augmentation	0.69	0.38	0.49	0.663
		0.83	0.95	0.88	
	SMOTE	0.62	0.5	0.56	0.704
		0.85	0.9	0.88	
	Positive augmentation	0.54	0.63	0.58	0.73
		0.88	0.83	0.85	
	4× augmentation	0.59	0.54	0.56	0.71
		0.86	0.88	0.87

**Table 6:** Comparison of accuracy, recall, F1 score, and AUC of the methods on test data by the deep network based on four data augmentation methods

Loss function	Data augmentation	Precision	Recall	F1	AUC
Cross entropy	Without augmentation	0.76	0.72	0.74	0.821
		0.91	0.93	0.92	
	SMOTE	0.58	0.82	0.68	0.813
		0.93	0.81	0.87	
	Positive augmentation	0.67	0.75	0.81	0.815
		0.92	0.88	0.90	
	4× augmentation	0.69	0.77	0.72	0.827
		0.92	0.89	0.91	
Focal loss	Without augmentation	0.76	0.73	0.74	0.828
		0.91	0.93	0.92	
	SMOTE	0.52	0.82	0.64	0.79
		0.93	0.76	0.84	
	Positive augmentation	0.74	0.72	0.73	0.817
		0.91	0.92	0.91	
	4× augmentation	0.7	0.74	0.72	0.821
		0.9	0.9	0.91	

Nonetheless, the network is able to attain high AUC 0.821 with an accuracy 0.7, recall 0.74 and F1 score of 0.72 on the abnormal data, 0.9, recall 0.9, and F1 score of 0.91 on the normal data for testing data, respectively, with the following confusion matrix given in Table 7.

**Table 7:**
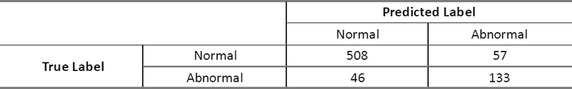
Results of the performance of our network method

**Fig. 8:**
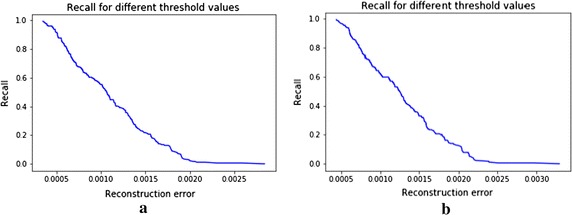
Recalls for different thresholds in CSDAE (**a**) with noise factor 0.01 and (**b**) noise factor 0.05

We can notice  the changes in precision and recall for different threshold values on reconstruction error from the CSDAE with noise factor 0.01 and 0.05 as shown in Figs. 7, 8. From Table 4, we see that the MSE of reconstruction error with mean 0.0009, std 0.00037 on the CSDAE with noise factor 0.01, as well as the MSE of reconstruction error with mean 0.00117 and std 0.00045 with noise factor 0.05. Hence, our CSDAE is a well- trained autoencoder for the normal case. We can screen the abnormal case from the CSDAE if its reconstruction error is above specific threshold value.

Now, two metrics are given for the final decision, and we can combine these two metrics to one score using the max area under the ROC curve (AUC).

6 $$\widehat{\beta }^{AUC} = \mathop {arg max}\limits_{{\begin{array}{*{20}l} {0 < T < T_{mean} } \\ {0.5 < P < 1} \\ \end{array} }} \left( {\widehat{AUC}(b)} \right),$$

where $T$ is the threshold, $T_{mean}$ is the average construction error of CSDAE, and P is the predicted probability from our CNNs. The confusion matrix of the performance of our method with max AUC for testing data is given in Table 8.

**Table 8:**
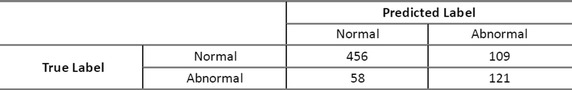
The results of the performance of our method with max AUC

Finally, we can compute the three levels pertaining to the image by voting on these different classifiers. For the testing data, we can obtain 395 normal cases, 88 abnormal cases, and 261 uncertainty case. Comparing with the ground truth, we can achieve precision of 98.7% (390/395) on normal cases and 94.3% (83/88) on abnormal cases for testing data. It is good to separate these images into three different levels with a total precision of 97.9% based on the normal and abnormal status levels. Achieving 100% of accuracy in CXR abnormality classification, to the best of our knowledge, does not exist as there is always possibility for false positive rate. We propose a method that has high detection rate and low false positive. In order to reduce false positives, this article proposes a variety of indicators and optimizes these indicators based on maximizing AUC. The results of this paper show that, the accuracy rate in our sample classified as normal is 98.7%, and the accuracy of classification as abnormality is 94.3%. In this way, we can divide the entire data set into three categories. This allows senior doctors to focus on high-risk suspected abnormal chest radiographs, mid-level physicians focus on uncertain categories, and lower-level medical experts can pay more attention to the suspected normal samples. Eventually, these experts can spend more time focusing on the high-risk chest radiographs. On the one hand, this greatly reduces the workload of the expert team, and correcting potential misdiagnosis. On the other hand, it can enable patients to receive timely medical treatment as a result of the time-to-diagnosis that is saved.

## Discussion

Many methods have been proposed to perform CXRs classification task, such as [21, 34]. Shin et al. [34, 35] used a CNN to detect specific diseases in CXRs images and achieved precision of 0.776 and recall of 0.76 based on the normal case, and then precision 0.556, recall 0.577 based on the nodule. Tataru et al. [21] attempted to classify a chest X-ray as normal versus abnormal in order to assist primary care physicians and radiologists to move more quickly and efficiently rather than render radiology obsolete with accuracy of 0.8 and F1 score of 0.66. However, our method yields an accuracy of 0.7, recall of 0.74, and F1 score of 0.72 on the abnormal data, while it achieves an accuracy of 0.9, recall of 0.9, and F1 score of 0.91 on the normal data. Yet, we cannot entirely compare these methods because of the different classification tasks and the databases. We only deal with the grade diagnosis for physical examination. The contribution of this paper is based on the fact that it proposes multiple new metric indices. Combining predictors for classification using area under the ROC curve is the proposed solution for this task. We find that, the final decision is related to the threshold from reconstruction error and the probability value.

## Conclusion

In this paper, we present an effective framework that learns and detects diseases from the patients’ CXRs based on the four public datasets. Furthermore, we introduce an approach to classify image levels by summarizing the hyper classifiers outputs and reconstruction error. Different metrics are used in this paper to classify the image levels. We combine the multiple classifiers using AUC in order to guarantee high confidence. Not only can this computer aided triaging system classify and detect disease in images, but also it can even compute the different image levels with promising results. Compared to existing methods, our method yields high accuracy, recall, and F1 score for the abnormal and normal datasets. Note that, in this research, our preliminary results cannot justify our proposed method to be fully adopted for an entirely automated chest screen system in clinical practice. However, this technique can partially help in the classification of normal versus abnormal CXRs and provide the physicians and radiologists with valuable information to significantly decrease time-to-diagnosis. To increase the performance of our method, we plan to build a big dataset for training intermediary deep network from clinical data in our future work. In addition, implementing an end-to-end learning model can be promising and may achieve high performance via optimally determined parameters.
